# The Place of Closed Mitral Valvotomy (CMV) Procedure in the Modern Era: 20 Years Single Center Experience.

**DOI:** 10.1186/1749-8090-10-S1-A319

**Published:** 2015-12-16

**Authors:** Ram Chandra Sherawat, Sunil Dixit, RK Yadav, Anil Sharma

**Affiliations:** 1 CTVS Department, Sawai Man Singh Medical College, Jaipur, Rajasthan, India

## Background/Introduction

The management of RHD with MS varies depending on severity of disease, availability of expertise and resource; however in a facility deprived country with low economic status closed mitral valvotomy remain the standard palliative treatment. CMV is indicated in patients with pure, non-calcific isolated MS with normal sinus rhythm.

## Aims/Objectives

The aim of this study was to evaluate the clinical status of patients with mitral stenosis following closed mitral valvotomy. About 96%, of the patients were in good health. These results suggest that there is still a good place for closed mitral valvotomy in carefully selected cases.

## Method

The suitable patients were selected according to echocardiography criteria: (1) pliable anterior mitral leaflet, (2) absence of significant mitral subvalvular disease, (3) absence of significant calcification, and (4) the mitral valve orifice area less than (1.1 cm^2^). The indications also include asymptomatic women of child bearing age with mitral valve areas of ~1.2 cm^2^.

## Results

In-hospital mortality was 0.5%. Cardiac failure with significant MR was the main cause of early death, and no postoperative peripheral embolism occurred in cases done after TEE and occurred in 0.5% cases done without TEE. Freedom from thromboembolism was 99.0 +/- 0.5% at 20 years. Operative results were satisfactory in most patients, and severe mitral incompetence was seen only in hundred cases in which 15 cases converted to open heart and remaining treated with medical treatment in which we lost 12 patients. Reoperation was performed in 1110 patients (25.57%). The mean interval between CMV and reoperation was 141.1 +/- 60.8 months (range: 1-240 months).

Hundred patients were reoperated for moderate or severe mitral regurgitation, 990 for mitral restenosis, and twenty for mixed mitral valve disease (stenosis and regurgitation). Freedom from reoperation after CMV was 81.4 +/- 1.3% at 10 years, 74.42 +/- 2.1% at 20 years. Cox regression analysis indicated that impaired functional capacity, reduced mitral valve area, gradual increase in left atrial diameter and postoperative mitral insufficiency increased the reoperation rate after CMV.

## Discussion/Conclusion

CMV represents a satisfactory technique in terms of lower cost, high efficacy, simplicity and reproducibility.

